# Transcriptomic changes during regeneration of the central nervous system in an echinoderm

**DOI:** 10.1186/1471-2164-15-357

**Published:** 2014-05-12

**Authors:** Vladimir S Mashanov, Olga R Zueva, José E García-Arrarás

**Affiliations:** 1 Department of Biology, University of Puerto Rico, PO Box 70377, PR 00936-8377 San Juan, USA

**Keywords:** Transcriptome, RNA-seq, Gene expression, Regeneration, Echinoderm, Nervous system, Transcription factors, Injury, Extracellular matrix

## Abstract

**Background:**

Echinoderms are emerging as important models in regenerative biology. Significant amount of data are available on cellular mechanisms of post-traumatic repair in these animals, whereas studies of gene expression are rare. In this study, we employ high-throughput sequencing to analyze the transcriptome of the normal and regenerating radial nerve cord (a homolog of the chordate neural tube), in the sea cucumber *Holothuria glaberrima*.

**Results:**

Our de novo assembly yielded 70,173 contigs, of which 24,324 showed significant similarity to known protein-coding sequences. Expression profiling revealed large-scale changes in gene expression (4,023 and 3,257 up-regulated and down-regulated transcripts, respectively) associated with regeneration. Functional analysis of sets of differentially expressed genes suggested that among the most extensively over-represented pathways were those involved in the extracellular matrix (ECM) remodeling and ECM-cell interactions, indicating a key role of the ECM in regeneration. We also searched the sea cucumber transcriptome for homologs of factors known to be involved in acquisition and/or control of pluripotency. We identified eleven genes that were expressed both in the normal and regenerating tissues. Of these, only *Myc* was present at significantly higher levels in regeneration, whereas the expression of *Bmi-1* was significantly reduced. We also sought to get insight into which transcription factors may operate at the top of the regulatory hierarchy to control gene expression in regeneration. Our analysis yielded eleven putative transcription factors, which constitute good candidates for further functional studies. The identified candidate transcription factors included not only known regeneration-related genes, but also factors not previously implicated as regulators of post-traumatic tissue regrowth. Functional annotation also suggested that one of the possible adaptations contributing to fast and efficient neural regeneration in echinoderms may be related to suppression of excitotoxicity.

**Conclusions:**

Our transcriptomic analysis corroborates existing data on cellular mechanisms implicated in regeneration in sea cucumbers. More importantly, however, it also illuminates new aspects of echinoderm regeneration, which have been scarcely studied or overlooked altogether. The most significant outcome of the present work is that it lays out a roadmap for future studies of regulatory mechanisms by providing a list of key candidate genes for functional analysis.

## Background

Echinoderms constitute a phylum of marine invertebrates closely related to chordates. Their phylogenetic position as a non-chordate deuterostome phylum combined with the ability to regenerate various body parts makes them a valuable group, which can give unique insights into fundamental issues of regenerative biology and provide new clues to finding better treatment of human conditions. Recent analyses of cellular events underlying post-traumatic regeneration in echinoderms (reviewed in [1,2]) identified interesting parallels with corresponding processes in regeneration-competent vertebrates, pointing to reparative mechanisms that might have been evolutionary preserved throughout Deuterostomia and could potentially be re-activated in poorly regenerating vertebrates. However, the paucity of genomic and transcriptomic information has precluded major progress in understanding key regulators of regeneration at the molecular level [3,4].

**Figure 1:**
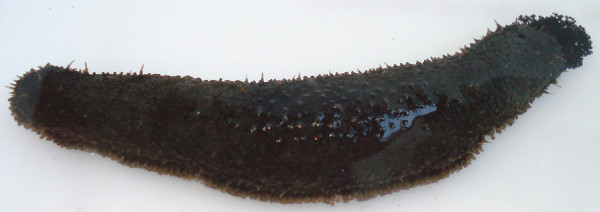
**The model organism used in this study: *Holothuria glaberrima* Selenka, 1867 (Echinodermata: Holothuroidea).**

**Figure 2:**
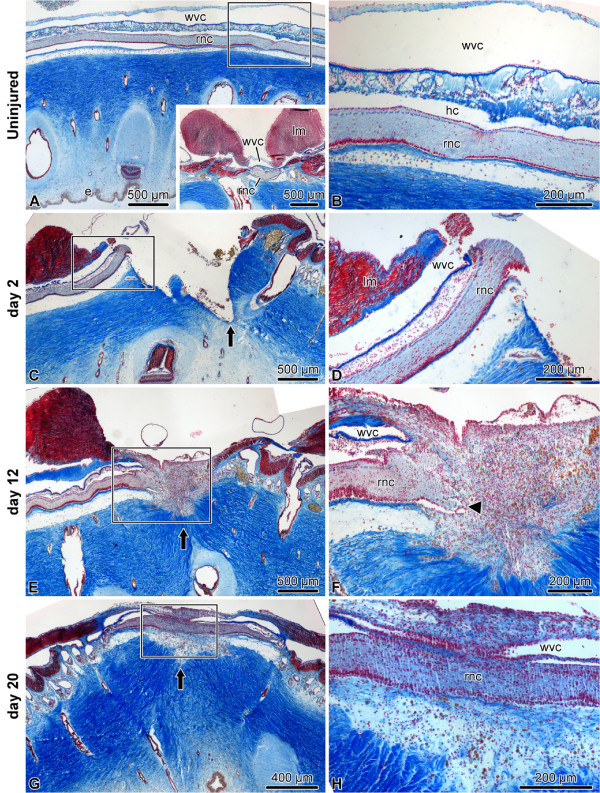
**Histological organization of the radial organ complex in non-injured (A, B) and regenerating animals on day 2 (C, D), 12 (E, F), and 20 (G, H) post injury.** All micrographs are longitudinal paraffin sections, except for A insert, which is a cross section. All sections were stained with Heindenhein’s azan. The right column **(B, D, F, H)** shows high magnification views of the boxed areas in the corresponding images of the left column (**A, C, E, G,** respectively). e, epidermis; hc, hyponeural canal; lm, longitudinal muscle; rnc, radial nerve cord; wvc, water-vascular canal. Arrows show the location of the plane of injury. The arrowhead in **F** shows the growing tip of the regenerating radial nerve cord.

The body of an echinoderm has radial organization and is composed of multiple (usually five) units, which are arranged as sectors or rays around the central axis connecting the mouth and the anus. Each of these radial units is supplied with a set of major organs, which in sea cucumbers (Figure 1) include a radial nerve cord (a homolog of the chordate neural tube [5]), a canal of the water-vascular system, and a longitudinal muscle band (Figure 2A, B).

Our previous research [1,6-8] showed that following a transverse cut, the injured organs of the radial organ complex on either side of the wound start growing across the wound gap and eventually reconnect to restore the anatomical continuity (Figure 2). The newly regenerated structures then completely re-acquire their normal tissue architecture and resume their functions. Radial nerve cord regeneration involves extensive dedifferentiation of radial glial cells in the vicinity of the injury. These dedifferentiated glial cells play the key role in subsequent regeneration through extensive proliferation, ECM invasion, and differentiation into new neurons and glial cells. The newly produced neurons are thought to be functionally integrated into the CNS circuitry, as they survive for extended periods of time and form typical synaptic connections [1,7].

The main goal of the present study is to help understand the molecular basis underlying the extensive regenerative capacity of the central nervous system in the sea cucumber *Holothuria glaberrima* by providing an outline of the transcriptomic landscape and thus identifying possible directions of future research. To this end, we used deep RNA sequencing on both the 454 and Illumina platforms to analyze changes in the transcriptome that occurred on day 2, day 12, and day 20 after injury. These time points were chosen based on our previous studies of cellular events in the regenerating radial nerve cord of *H. glaberrima*[1]. Day 2 post-injury (Figure 2C, D) is the early post-injury phase of extensive dedifferentiation of radial glia, axonal degeneration, and programmed cell death in the injured radial nerve. Day 12 post injury (Figure 2E, F) corresponds to a period of active growth across the wound gap; dividing cells are most abundant at this stage. Day 20 (Figure 2G, H) post injury is a late regeneration phase, when the two growing regenerates have restored their anatomical continuity and started to resume their typical histological architecture [1,7]. The present study provides first insights into gene expression changes that underlie these previously described cellular events.

## Results and discussion

### Sequencing and assembly: technical information about the assembly

**Figure 3:**
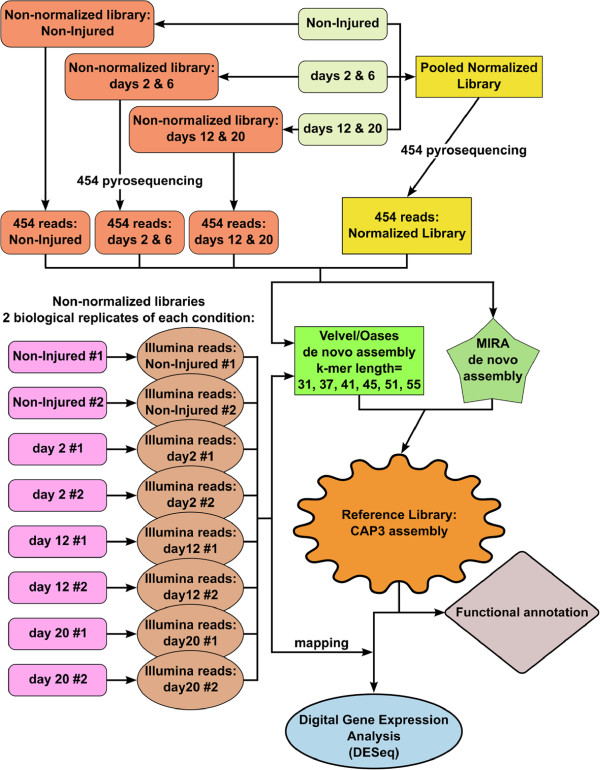
Summary of major steps involved in library preparation, sequencing, assembly, and annotation workflow.

The present study was performed on the brown rock sea cucumber *Holothuria glaberrima* Selenka, 1867 (Echinodermata: Holothuroidea), an established model organism in echinoderm regenerative biology. We injured the radial nerve cord and the surrounding tissues, including the body wall connective tissue, water-vascular canal, and the longitudinal muscle band (Figure 2C), by performing a single transverse cut through these organs in the mid-ventral ambulacrum at about the mid-body level. We then harvested the regenerating tissues on day 2, day 6, day 12, and day 20 post-injury, as well as non-injured radial nerve cord and used the samples for sequencing library preparation. The major steps involved in our sequencing and data analysis pipeline are outlined in Figure 3.

The high-throughput transcriptome sequencing yielded a total of 2,428,740 Roche 454 reads with the average/modal length of 595/546 bases and 331,931,211 single-end Illumina reads (75 bp) (Table 1). We first combined the reads from all libraries to assemble a reference transcriptome for annotation purposes. Prior to the de-novo assembly step, we subjected the raw reads from both sequencing platforms to a rigorous cleaning/clipping procedure (see Methods for details) leaving 1,620,029 Roche 454 reads (with the average/modal length of 534/523 bases, respectively) and 246,993,807 Illumina reads (with the average/modal length of 57/61 bases, respectively) for further analysis (Table 1). All cleaned 454 reads were assembled with MIRA (Figure 3) into 125,828 contigs with the mean length of 676 bp and N50 (i.e., the contig length such that 50% of the total assembled sequence is contained in contigs of this size or longer) of 720 bp (Table 2). Illumina reads were assembled with Velvet and Oases (Figure 3). De Bruijn graph assemblers, such as Velvet/Oases, are known to be sensitive to the value of parameter k, which is under the control of the user. It has been shown that the use of a single k-mer length may result in suboptimal de novo transcriptome assemblies [9]. For a given assembly, the best k-mer value depends on the coverage depth (which is distributed over wide ranges in non-normalized libraries), the sequencing error rate, and the complexity of the transcriptome. Changing the k-mer value in either direction (i.e., increasing or decreasing) is associated with both pros and cons. Using higher k-mer values increases specificity and leads to a better assembly of highly expressed transcripts, whereas lower k-mer values improves sensitivity and allows better reconstruction of weakly expressed transcripts [9,10]. Therefore, seven separate assembly runs were performed with different k-mer length (ranging from 31 to 55). They yielded between 50,903 and 237,177 contigs (assemblies with the higher k-mer value producing the lower number of contigs), with the mean length of 674 – 800 bp, the maximum length of 16,096 – 27,112, and N50 ranging between 1,377 and 1,555 (Table 2). Then all MIRA and Velvet contigs were pooled together and used as an input for the final assembly run with the CAP3 program (Figure 3), which produced 70,173 contigs with dramatically improved mean length (1,558 bp) and N50 (2,767 bp) and the maximum length of 27,089 bp (Table 2). We refer to this final set of 70,173 contigs (available in the LabArchives notebook [11]) as a reference library, which represents the transcriptional diversity in the normal and regenerating radial organ complex of the sea cucumber *H. glaberrima*, and which we used for functional annotation and read mapping (Figure 3). Previously, 53 of these contigs were found to correspond to 36 long terminal repeat (LTR) transposons. Homology analysis and expression pattern of these mobile genetic elements were characterized in detail elsewhere [12,13] and, therefore, will not be covered in this study.

**Table 1:** Summary statistics of sequencing runs and read processing

	**Total number**	**Total number**	**Mean read**	**Modal (most frequent)**	**Read length**
	**of reads**	**of bases**	**length**	**read length**	**range**
**Total Raw 454 reads**	2,428,740	1,444,989,971	595	546	49 – 1,201
**Total Cleaned 454 reads**	1,620,029	864,494,207	534	523	60 – 994
Raw Illimina Norm #1	41,062,198	3,079,664,850	75	75	75
Raw Illimina Norm #2	41,396,915	3,104,768,625	75	75	75
Raw Illimina d2 #1	40,461,887	3,034,641,525	75	75	75
Raw Illimina d2 #2	42,713,246	3,203,493,450	75	75	75
Raw Illimina d12 #1	42,485,405	3,186,405,375	75	75	75
Raw Illimina d12 #2	42,718,652	3,203,898,900	75	75	75
Raw Illimina d20 #1	38,333,067	2,874,980,025	75	75	75
Raw Illimina d20 #2	42,759,841	3,206,988,075	75	75	75
**Total Raw Illumina**	**331,931,211**	**24,894,840,825**	**75**	**75**	**75**
Cleaned Illimina Norm #1	30,668,029	1,760,422,119	57	61	32 – 61
Cleaned Illimina Norm #2	29,685,397	1,717,566,430	58	61	32 – 61
Cleaned Illimina d2 #1	28,982,010	1,648,498,857	57	61	32 – 61
Cleaned Illimina d2 #2	30,382,668	1,721,162,425	57	61	32 – 61
Cleaned Illimina d12 #1	33,755,449	1,947,272,365	58	61	32 – 61
Cleaned Illimina d12 #2	33,893,212	1,956,101,048	58	61	32 – 61
Cleaned Illimina d20 #1	29,670,665	1,697,625,162	57	61	32 – 61
Cleaned Illimina d20 #2	29,956,377	1,693,992,519	57	61	32 – 61
**Total Cleaned Illumina**	**246,993,807**	**14,142,640,925**	**57**	**61**	32–61

**Table 2:** Summary statistics for the intermediate and final assembly steps

	**Total number**	**Total number**	**Mean**	**Length**	**N50**
	**of contigs**	**of bases**	**length, nt**	**range, nt**	
Normalized and non-normalized 454 reads combined	125,828	85,117,028	676	100 – 20,566	720
Velvet (k-mer length=31)	237,177	162,204,944	684	100 – 16,096	1,555
Velvet (k-mer length=37)	188,901	127,287,870	674	100 – 27,112	1,496
Velvet (k-mer length=41)	158,033	111,038,497	703	100 – 27,083	1,554
Velvet (k-mer length=45)	133,348	93,061,666	698	100 – 25,388	1,464
Velvet (k-mer length=51)	87,923	63,748,786	725	100 – 23,585	1,377
Velvet (k-mer length=55)	50,903	40,710,596	800	100 – 23,018	1,386
**Reference Assembly (CAP3)**	**70,173**	**109,360,184**	**1,558**	**100 – 27,089**	**2,767**

To assess the accuracy of our sequencing and assembly pipeline, we re-sequenced 16 randomly selected contigs from the reference library using Sanger technology (Table 3). In total, 34,708 nt of 44,096 nt (∼79%) were analyzed by re-sequencing. The overall error rate (determined as a percentage of mismatch between Sanger sequencing and next-generation RNA-seq) was 0.19%, with insertions, deletions, and substitutions affecting 29 nt, 16 nt, and 20 nt, respectively. Eleven of the resequenced transcripts had a complete open reading frame (ORF), in four contigs both the 5’ and 3’ ends of the ORF were incomplete, in one contig, only the 5’ end was missing, and one contig was likely a non-coding RNA. One of the 14 ORF-containing contigs had a frameshift error.

**Table 3:** Validation of assembled contigs by re-sequencing using Sanger technology

**ContigID**	**Best blastx**	**ORF complete?**	**Frameshifts**	**Contig length, bp**	**Checked by Sanger**	**Insertions**	**Deletions**	**Substitutions**	**% Discrepancy**
	**match**		**in ORF**		**sequencing, bp**				
Contig3756	CENPS	yes	no	665	564	8	0	1	1.60%
Contig7869	Myc	yes	no	3,268	2,922	0	0	0	0.00%
Contig54848	HuD/Elav	yes	1	4,404	3,431	9	2	3	0.41%
Contig19303	Lgr5	both 5’ and 3’ ends are missing	no	1,510	1,510	0	0	0	0.00%
Contig40997	NeuroD	both 5’ and 3’ ends are missing	no	745	470	0	1	2	0.64%
Contig1064	NF1A	yes	no	5,279	3,825	3	9	1	0.34%
Contig32193	NvProtien1	5’ end is missing	no	2,411	1,986	0	1	0	0.05%
Contig51632	Oct	both 5’ and 3’ ends are missing	no	1,947	1947	0	0	0	0.00%
Contig46302	Piwi	yes	no	3,430	2,929	0	0	3	0.10%
Contig67140	Sox	yes	no	3,096	2,889	2	0	0	0.07%
Contig20197	unknown	NA	NA	1,339	1,252	2	0	0	0.16%
Contig68289	Wnt3	yes	no	2,561	990	2	0	2	0.40%
Contig50192	DCLK	yes	no	3,152	2,474	0	0	3	0.12%
Contig8186	Lhx	yes	no	5,430	3,041	0	0	5	0.16%
Contig3071	FoxJ1	yes	no	2,523	2,404	2	3	0	0.21%
Contig10179	Klf	yes	no	2,336	2,074	1	0	0	0.05%
			**TOTAL:**	**44,096**	**34,708**	**29**	**16**	**20**	**0.19%**

In order to determine the proportion of the contigs in the reference library, which corresponded to known proteins, we performed a BLASTX search against publicly available protein databases. In the first round of search, the sequences were matched against the Swissprot database. The contigs, which did not produce hits passing the significance threshold corresponding to anE-value < 1e-6 were subsequently subjected to a second round of BLASTX search against the non-redundant (nr) NCBI protein database with the same threshold. Overall, 24,324 (or 33.66%) contigs had significant BLAST hits. The results of the BLAST analysis are listed in Additional file 1.

We also annotated the sea cucumber transcriptome by performing reciprocal best BLAST hit analysis (with a threshold e-value < 1e-6) versus the NCBI’s collection of the sea urchin *Strongylocentrotus purpuratus* predicted protein sequences [14], the echinoderm species whose genome has been most thoroughly characterized so far. There were 23,637 one-way BLASTX matches between the *H. glaberrima* contigs and the *S. purpuratus* proteins and 19,869 one-way TBLASTN matches between the sea urchin proteins and the contigs of our transcriptome. The number of reciprocal blast matches (putative orthologous sequences) was 8,577. The results of this analysis are listed in Additional file 2.

We, obviously, do not expect all our 70,173 contigs to represent individual sea cucumber genes. It is impossible to give an exact answer on the number of unique genes represented by de novo assembled contigs without having complete genomic information. Thus, we can only provide an educated guess here. The genome of the sea urchin *S. purpuratus*[15] encodes ∼23,300 genes, of which ∼7,000 are presumed orthologs of mammalian genes. In the course of our analysis, a similar number (8,522) of unique mouse proteins showed significant similarity to sequences contained in the assembled transcriptome of *H. glaberrima*. If we assume the same level of genomic similarity between mammals and each of the two echinoderm species, the similar number of “mammalian genes” observed in the sea urchin genome and the sea cucumber transcriptome would imply that (i) sea cucumbers have roughly the same number of genes as sea urchins (∼23,000), and (ii) most of these genes are represented in our reference library constructed from mRNA samples from non-injured and regenerating animals. If the above reasoning is correct, there is a ∼3× redundancy in our assembly.

A similar redundancy ratio (∼2.4×) can be obtained, if we consider that, when blasted against the reference mouse proteome, 20,619 of 70,173 contigs of the sea cucumber reference library matched 8,522 unique mouse proteins. Some of this redundancy is part of the “natural” variation that is expected from differences in mRNA processing (such as splicing). In fact, when manually inspecting assembled contigs, we saw polymorphic transcripts, which were especially common among retroelements. Additional variation can be due to the limitations of the sequencing techniques and/or de novo assembly programs.

### Differential gene expression in radial organ complex regeneration

**Figure 4:**
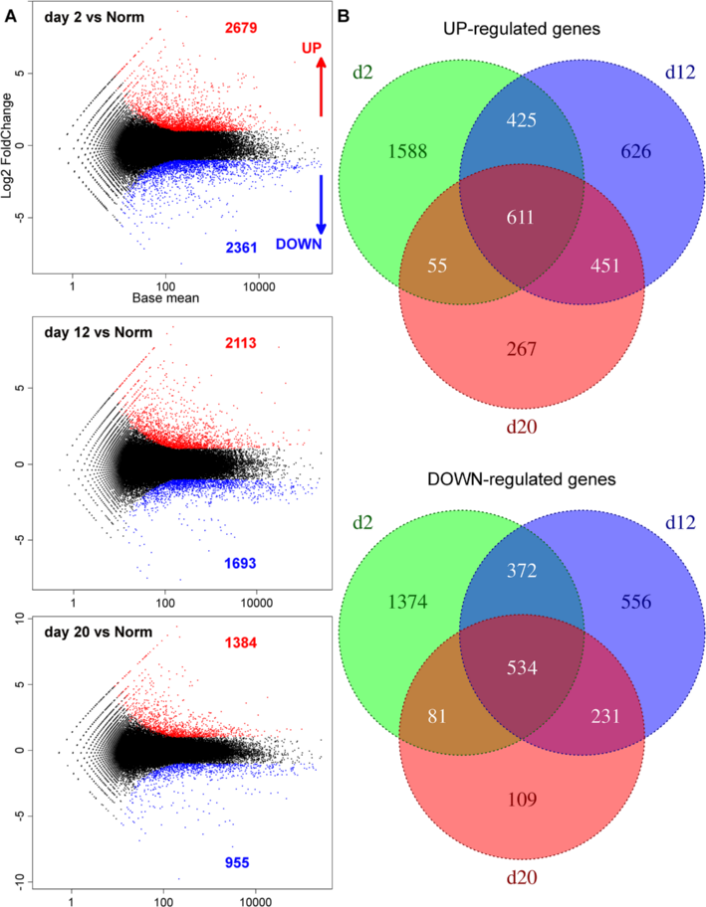
**Differential gene expression analysis of the regenerating radial organ complex. (A)** DESeq scatter plots showing comparisons of gene expression levels between regenerating and uninjured animals. Each dot represents the mean expression level for a given contig. Differentially up-regulated contigs (with false discovery rate < 0.001 and a threshold log2 fold change of ±1) are shown in red and differentially down-regulated contigs are shown in blue. Numbers above or below the plots refer to the total number or up- or down-regulated contigs, respectively, at a given time point of regeneration. **(B)** Venn diagrams showing the numbers of common and distinct differentially up-regulated (top) and down-regulated (bottom) genes at different time points of regeneration.

To characterize differentially regulated genes involved in regeneration, we mapped the reads from each of the eight Illumina libraries (two biological replicates for each of the four conditions — non-injured animals and regenerating organs on days 2, 12 and 20 post-injury) to the contigs of the reference library (Figure 3). Gene expression values in each pair of replicates showed high correlation (Pearson’s product-moment correlation coefficient r = 0.94 – 0.98, p < 2.2e-16) (Additional file 3). We then used the DESeq package [16] to identify sets of significantly up- and down-regulated genes in the regenerating tissues as compared with the intact animals (Figure 4). Upon imposing a threshold adjusted *P* value (*P *_*adj*_) of less than 0.001 and a threshold fold change of 2, we identified a total of 4,023 and 3,257 transcripts, which were differentially up-regulated and down-regulated, respectively. The distribution of unique and overlapping sets of the differentially expressed genes at different time points is summarized in Figure 4B. Note that the early post-injury phase (day 2) is characterized by the highest values of both the total number of differentially expressed transcripts and the number of unique up-regulated and down-regulated genes. On the other hand, in line with the general histological similarity between the late regenerates and uninjured radial organs (Figure 2A, B, G, H) [1,7], the samples analyzed on day 20 post-injury showed the lowest number of differentially regulated transcripts.

At all three time points after the injury, the majority (∼63-85%) of the significant differentially expressed contigs were up- or down-regulated between 2- and 4-fold relative to the normal animals (Additional file 4), suggesting that relatively small changes in transcript abundance of most genes are sufficient for regeneration to occur. Among the most extreme outliers in our differential expression analyses were sequences that were identified as retrotransposon-derived transcripts. Some of them showed an over 50-fold change in expression during regeneration. This unexpected finding prompted us to undertake a separate study of these mobile genetic elements that has already been published elsewhere [12].

**Figure 5:**
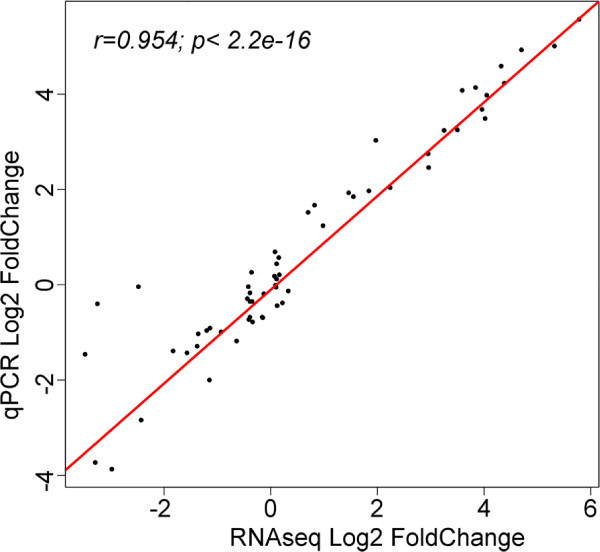
**Comparison of mRNA expression levels as determined by RNA-seq and qRT-PCR.** Comparisons were performed for 21 genes at three time points of regeneration relative to uninjured animals (for the list of genes and numerical values, see Additional file 5). The red line is the linear regression line.

To verify the validity of our approach and the accuracy of the large-scale digital gene expression assay, we selected 21 genes for analysis by quantitative real-time RT-PCR (qRT-PCR). This dataset also includes previously determined expression values for three LTR retroelements (*Gypsy-1_Hg, Gypsy-2_Hg,* and *Gypsy-20_Hg*) characterized earlier elsewhere [12]. Our goal was to validate the sensitivity of the assay at different levels of transcript abundances. Therefore, we chose not only highly over-expressed or down-regulated genes, but also genes with moderate fold change and genes, whose transcript abundance remained stable throughout the experiment. We found highly significant strong positive correlation between the RNA-seq and qRT-PCR data (Pearson’s r=0.954, p <2.2e-16) (Figure 5). The corresponding fold change ratios (relative to the uninjured animals) for both quantification techniques are listed in Additional file 5. This correlation analysis suggests that our RNA-seq data reliably represent relative changes of mRNA levels in the regenerating tissues.

### Functional annotation of differentially expressed genes at different time points of regeneration

**Figure 6:**
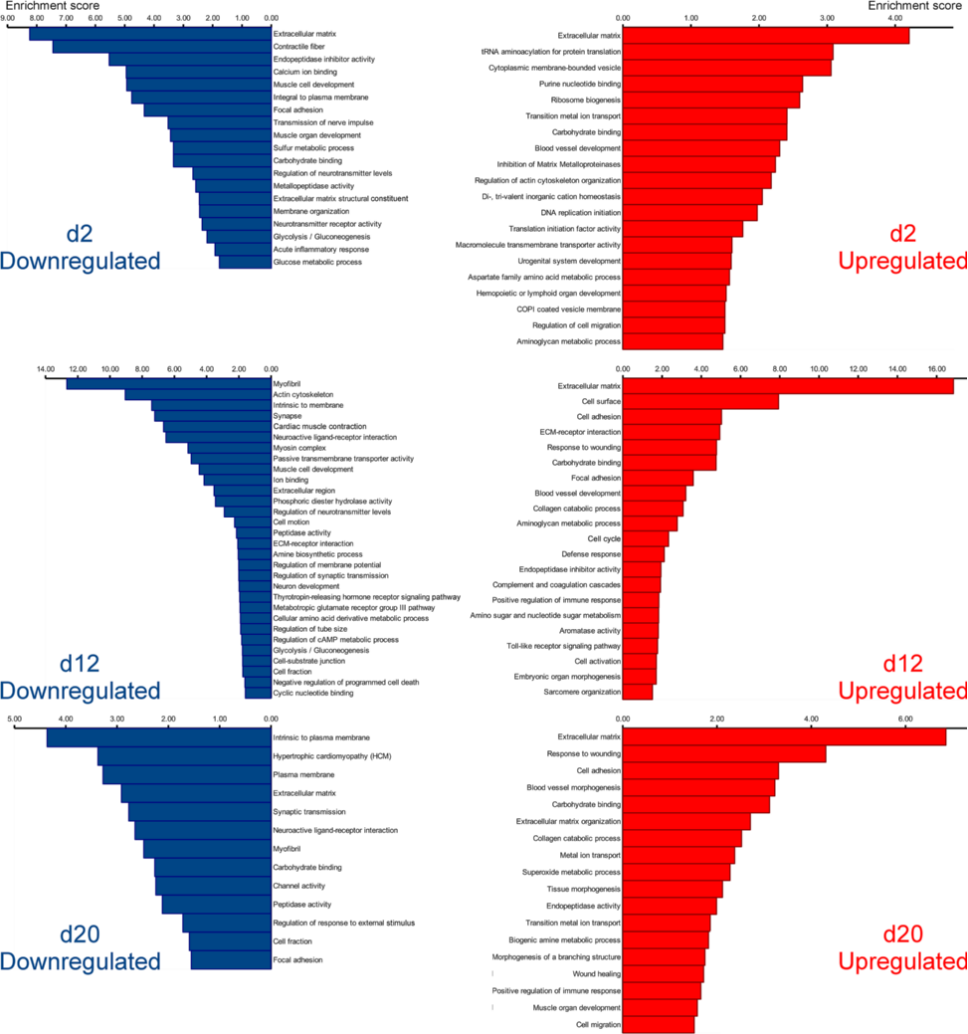
**Diagram showing clustering of functional annotation terms associated with differentially expressed genes during the radial organ complex regeneration.** Differentially expressed genes that are up-regulated (right column) or down-regulated (left column) at a given time point of regeneration were analyzed for significantly enriched functional annotation terms, which were clustered using DAVID. Functional annotation clusters with enrichment scores (in negative log scale) > 1.3 are shown. The corresponding detailed data resulting from DAVID output are listed in Additional file 6).

From the biological perspective, it is important to know which processes predominate or become suppressed at different time points during regeneration. For functional annotation, we employed an approach, which was conceptually similar to that described by Stewart et al. [17] for the analysis of the limb blastema transcriptome in axolotl. In order to gain insight into the biological meaning behind the large numbers of differentially regulated transcripts, we analyzed the lists of genes that showed significant changes in expression levels (with the cut-off set at ±1 log2 fold change, and P _*adj*_< 0.001) with DAVID v6.7 [18,19] to determine enriched functional categories. To be able to use this tool, we had to match the contigs resulting from our de novo assembly of the sea cucumber transciptome to genes of some well-annotated model organism. We thus annotated the assembled transcriptome of *H. glaberrima* against the non-redundant reference proteome of the mouse. Our assembled contigs matched to 8,522 unique mouse genes at the BLASTX cut-off value of 1e-6. This entire list of annotated contigs representing both uninjured and regenerating animals was submitted to DAVID along with the lists of differentially expressed genes and used as a custom reference background. Functional annotation clustering was performed using Gene Ontology (GO_TERM_BP_FAT, GO_TERM_CC_FAT, GO_TERM_MF_FAT) and pathway (KEGG, BIOCARTA, PANTHER) annotations. The functional clustering algorithm in DAVID groups together statistically enriched annotation categories if they share significant degrees of similarity in terms of associated gene members. The EASE score (modified Fisher exact p-value) threshold was set at 0.05. Functional annotation clusters with enrichment scores (geometric mean of EASE scores, in negative log scale) > 1.3 (which is equivalent to a non-log scale value of 0.05) were considered significant. The following major findings emerged from this analysis (Figure 6; Additional file 6). 1. Among the most enriched functional categories associated with both up-regulated and down-regulated genes at all time points were those related to synthesis and organization of the extracellular matrix (ECM) components, ECM remodeling, and interaction between cells and the extracellular matrix (see below for a detailed discussion).

2. Functional annotation terms associated with normal physiology, differentiation, and development of the nervous tissue were over-represented among the down-regulated genes on days 2 and 12 post-injury, and some of those annotation categories were also enriched among negatively regulated genes even as late as after 20 days post-injury. This observation correlates well with previously published morphological studies showing extensive dedifferentiation in the regenerating radial nerve at these time points [1,7]. Interestingly, annotation categories related to glycolysis were also enriched among the down-regulated genes on day 2 and 12.

Since our tissue samples, in addition to the radial nerve cord per se, also contained some adjacent tissues, including the contractile epithelium of the water-vascular canal and the coelomic myoepithelium of the body wall (see Methods), annotation terms associated with structural components of muscular tissue, as well as with muscle development and physiology, also appeared in the analysis. These terms were enriched in sets of down-regulated genes at all three time points of regeneration corroborating earlier observations of muscular dedifferentiation during body wall regeneration in sea cucumbers [6].

3. On day 2 post-injury, the up-regulated genes were associated with over-represented terms related to initiation of DNA replication and protein translation. On day 12, positively regulated genes were characterized by continued over-representation of terms associated with DNA synthesis and cell cycle. These data corroborate our cell proliferation assay, which suggested that the peak of cell division in the regenerating radial nerve cord occurs on days 8 thru 12 after injury [1]. On day 12, up-regulated genes are also enriched in annotation categories associated with activation of the innate immune response. The latter group of functional terms remains over-represented among up-regulated genes on day 20.

4. All three regeneration time points are characterized by over-representation of annotation categories associated with developmental morphogenesis, indicating involvement of ontogenic processes in regeneration.

**Figure 7:**
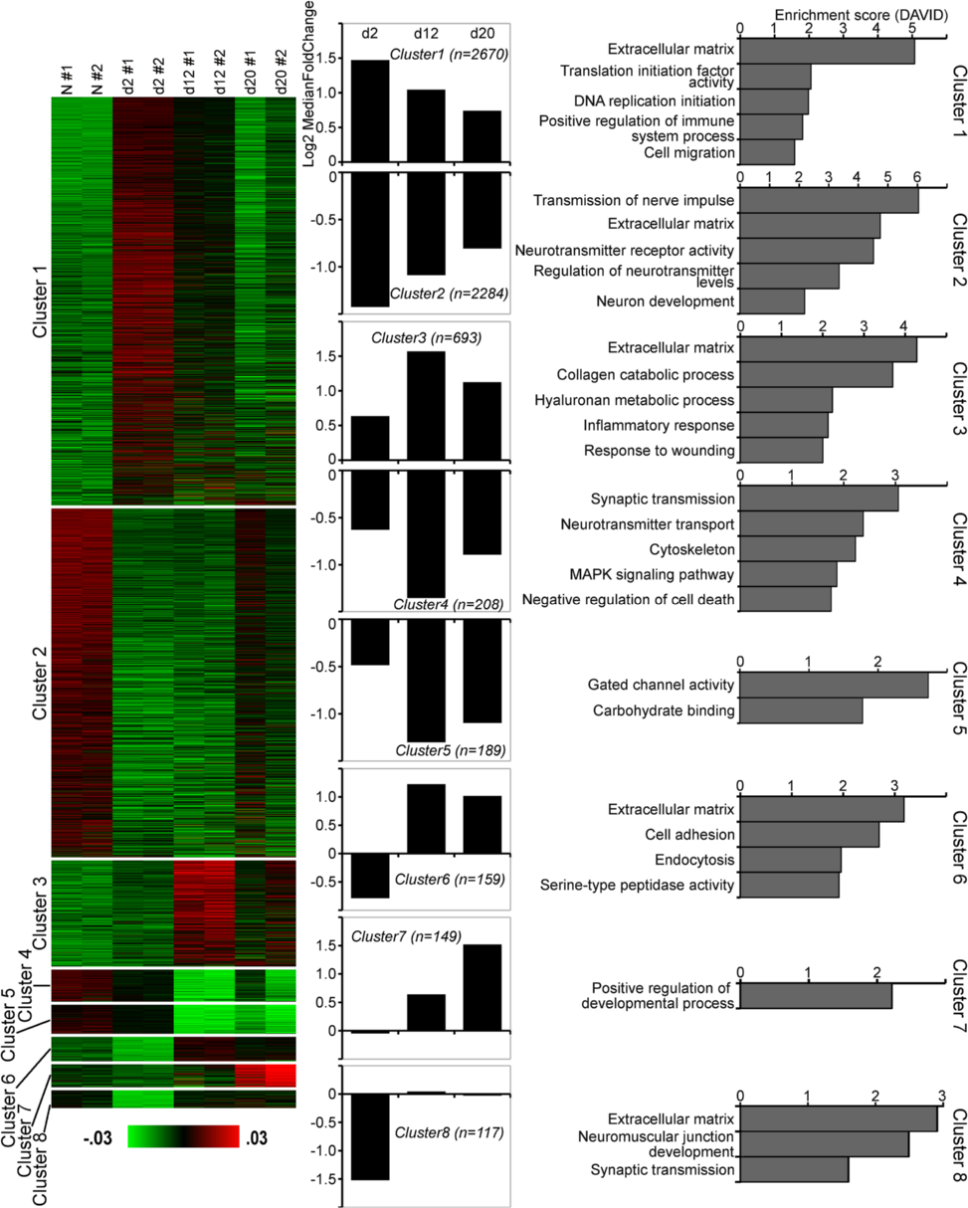
**Clustering of co-expressed genes.** The left row shows a heatmap produced by AutoSOME with clusters of co-expressed genes. Only eight clusters containing more than 100 genes are shown. The middle column shows median log2 fold-change in expression (vs uninjured animals) of the genes contained in each cluster. The clustered transcripts were analyzed with DAVID for significantly enriched functional annotation terms. The right column shows representative functional categories and their enrichment score.

In order to get further insight into mechanisms controlling gene expression in post-traumatic regeneration of the radial nerve cord, we performed clustering of differentially expressed genes using the unsupervised clustering algorithm implemented in the AutoSOME program [20] to identify sets of co-regulated genes with similar patterns of expression throughout the time course of regeneration. This gene co-expression analysis yielded eight distinct clusters with more than 100 genes in each (Figure 7; Additional file 7). In two of these clusters (clusters 1 and 3), the median gene expression in regenerating animals was consistently higher at all three regeneration stages when compared with uninjured animals. These genes were enriched in functional annotation terms related to the extracellular matrix, initiation of DNA replication and translation, positive regulation of immune response, wound healing, and cell migration. Three other clusters (clusters 2, 4, 5) contained genes, whose median expression at all three time points was lower that in normal animals. Associated enriched functional annotation terms were related to extracellular matrix, transmission of nerve impulse, regulation of neurotransmitter activity, muscle cell and neuron development, cytoskeleton, MAPK signaling pathway, negative regulation of cell death, gated channel activity, and carbohydrate binding. Clusters 6 and 7 contained genes, whose expression initially either remained unchanged or somewhat decreased at the early post-injury stage (day 2), but then was elevated during the growth (day 12) and late regeneration (day 20) phases. These genes were associated with positive regulation of developmental processes, extracellular matrix, cell adhesion, serine peptidase activity, endocytosis. Cluster 8 contained genes, which were markedly down-regulated at the early post-injury stage (day 2), but whose expression than returned to the normal levels as regeneration progressed. Enriched functional annotation terms associated with this cluster included extracellular matrix, neuromuscular junction development, and synaptic transmission.

### Putative regulation of groups of co-expressed genes by transcription factors (TFs)

**Figure 8:**
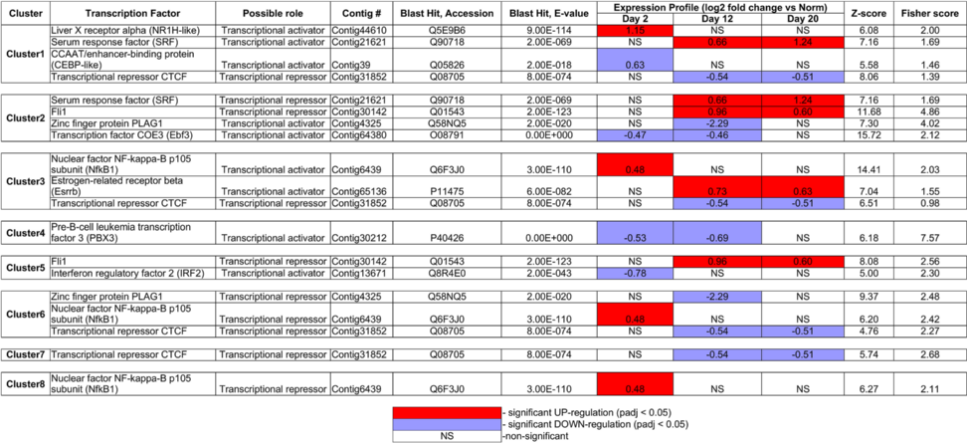
**Putative regulation of co-expressed transcripts by transcription factors (TFs).** Co-expressed genes contained in clusters identified with AutoSOME (Figure 7) were analyzed for over-representation of putative TF binding sites using oPOSSUM.

We then sought to get insight into which upstream transcription factors (TFs) may regulate coordinated expression of genes within each of the eight clusters identified above (Figure 7). Over-representation of transcription factor binding sites associated with co-expressed genes of each cluster was predicted using oPOSSUM software [21]. We considered only those of the predicted TFs whose significant (p < 0.05) changes in expression level correlated in time (at least at one time point) with changes in the median gene expression within the respective cluster. This analysis yielded 11 TFs, which putatively regulate regeneration-related genes (Figure 8) and therefore constitute good candidates for further functional analysis. It is worth noting that there was some overlap between the clusters. Thus, *CTCF* and *NfkB1* were associated with four and three clusters, respectively, and *SRF, Fli1,* and *PLAG1* were associated with two different clusters each. Some of the TFs (for example, *SFR, NfkB1*) were identified as functioning as both transcriptional activators and transcriptional repressors in different clusters.

Among these predicted 11 TFs, there are genes that have been previously implicated in post-traumatic processes and developmental processes in various organisms. For example, *NfkB1* is known to affect expression of broad range of downstream genes involved in various biological processes including immunity, differentiation, and programmed cell death. For example, elevated NfkB signaling has been previously shown to activate the Wnt signaling pathway and thus induce dedifferentiation of nonstem cells [22]. Another TF, *serum response factor (SRF)*, was shown to be one of the key genes involved in post-traumatic regeneration initiation in planarians [23]. In gastric ulcer healing, *SRF* promotes re-epithelialization and muscle regeneration through activation of cell migration and proliferation [24]. Moreover, *SRF* is also implicated in neuronal cell migration and axonal guidance through regulation of components of the actin cytoskeleton [25]. Still another gene, *CCAAT/enhancer binding protein (CEBP)* was suggested to be involved in regulation of the innate immune response during tissue injury repair [26].

Interestingly, the candidate TFs identified in this study included not only previously known regeneration-related genes, but also factors not previously known to act as regulators of post-traumatic tissue regrowth, such as, for example, *liver X receptor, Fli1, PLAG1, Ebf3, Esrrb*. The potential role of these genes in regeneration deserves further attention in future research.

### Discussion of selected functional gene groups

The preceding section provided an overview of unbiased functional characterization of the regeneration-associated genes at the global, transcriptome-wide level. Below, we further zoom in on certain groups of differentially expressed genes, which we picked from our database based on the results of the above analysis, prior biological knowledge, or both.

#### Cancer-related pathways

As revealed by DAVID analysis, the set of differentially expressed genes at the early post-injury stage (day 2) includes many known cancer-related genes (Additional file 6). For example, pathway mapping revealed differential regulation of Wnt receptors (*Fzd3* and *Fzd4*) and ligands (*Wnt9, Wnt2*, and *Wnt6*). Another notable observation is down-regulation of *survivin (Birc5)* (Additional file 8). This gene codes for a multifunctional protein, highly expressed in most human cancers, and implicated in both suppression of programmed cell death [27] and regulation of cell division [28-30]. We have previously shown that elevated expression of *survivin* in regenerating sea cucumber intestinal tissues correlated with low levels of apoptosis [31]. Reduced expression of this gene in the regenerating radial nerve cord may thus be at least in part explained as being associated with extensive programmed cells death in the vicinity of the injury [1].

Differential expression of oncogenes early in regeneration has been also reported for other model systems, including early limb blastema in axolotl [17] and can be explained by the fact that both regeneration and cancer progression are developmental processes that share a number of key mechanisms including cell division, programmed cell death, and differentiation.

#### ECM-related pathways

As mentioned above, among the most significantly over-represented pathways associated with differentially expressed genes at all three time points of the radial nerve cord regeneration were those related to the extracellular matrix (ECM) components and ECM-cell interactions (see, for example, Additional file 9 showing mapping of our RNA-seq data to the Focal Adhesion KEGG pathway). One example is significantly reduced expression of genes coding for basal lamina proteins on day 2 (*Col4a1, Lama4*), but up-regulation of the fibrillar collagens *Col5a1* and *Col11a2*. These data correlate well with previous observations of breakdown of the basal lamina during the early post-injury phase [7].

Of particular interest is concurrent up-regulation of both matrix metalloproteases (MMPs) and their inhibitors, TIMPs, in the regenerating radial organ complex. By breaking down components of the connective tissue matrix at the injury site and thus affecting cell migration and proliferation, epithelialization, differentiation, and apoptosis, MMPs are known to facilitate wound healing and regeneration [32-34]. However, up-regulation of TIMPs is as important for precise regulation of the regenerative processes, as they protect newly synthesized ECM matrix from degradation [32].

Proper interactions between cells and the ECM are essential both for correct tissue organization and signal transduction. Among the main cell adhesion molecules mediating these interactions are integrins [35-37]. In regeneration of the radial nerve in the sea cucumber, *integrin beta* and *integrin alpha-8* are significantly up-regulated (Additional file 9). Integrins affect cytoskeletal organization through Cdc42, a member of the Rho family of small GTPases [38,39]. Expression levels of the *Cdc42* transcript were consistently highly elevated in the sea cucumber radial nerve regeneration, suggesting a possible involvement of the integrin-Cdc42 pathway in control of ECM-cell interactions in this animal model.

Besides integrins, other cell adhesion molecules are also up-regulated during the early post-injury phase. These include selectins (Additional file 6). Interestingly, expression of selectins is known to be induced by NF-kB [40], which was putatively identified as one of the putative key transcription factors driving differential gene expression during the early post-injury stage of neural regeneration in our model (see above).

#### Pluripotency factors

**Figure 9:**
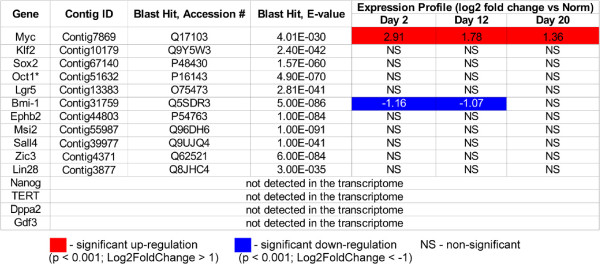
**Expression of pluripotency factors in regeneration of the radial organ complex in the sea cucumber *H. glaberrima*.** Summary of the digital expression assay and homology search against the UniProt database. Red and blue colors indicate significant (adjusted p < 0.001, more than two fold change in expression level) up-regulation or down-regulation, respectively, in the regenerating tissues as opposed to uninjured organs. NS, no significant changes in expression was observed. *Note that the gene designated as *Oct1* is included in this list because it is the only Oct homolog found in the *H. glaberrima* transcriptome. Likewise, *Oct1/2* is the only Oct in the sea urchin genome [44]. In mammals, there are a number of other Oct genes that perform various essential roles. One of them, Oct4 was used to induce pluripotency in mammalian somatic cells [41,45].

As has been documented by morphological studies [1,6,7], regeneration of the radial organs in sea cucumbers involves extensive dedifferentiation of specialized cells of the adult tissues in the vicinity of the injury. The dedifferentiation step endows these cells with increased proliferative potential, as well as with the ability to migrate and subsequently re-acquire their initial morphology or, more interestingly, transdifferentiate to other cell types. As this dedifferentiation phase is an indispensable component of the regenerative response, we asked if it requires elevated expression of factors known to be involved in acquisition and/or control of pluripotency. We, therefore, searched our assembled transcriptome for homologs of those genes and analyzed their expression values based on our RNA-seq read count data (Figure 9). Out of 15 factors analyzed, 11 were found to be expressed in both normal and regenerating tissues. Of those 11 genes, only the *Myc* homolog was expressed at significantly higher levels in regeneration. *Myc* genes have a wide variety of functions affecting the developmental potential of cells, including driving cells into the cell cycle, promoting the open state of chromatin, and DNA replication. *C-Myc* is one of the Yamanaka factors involved in the production of induced pluripotent cells [41] from specialized mammalian cells. During this reprogramming procedure, Myc represses differentiation-associated genes [42,43]. The functional role of Myc in echinoderm regeneration is a subject of ongoing research in our group.

To our surprise, we also found that expression of the *Bmi-1* homolog was significantly reduced in the regenerating tissues on days 2 and 12 post-injury. The remaining 9 genes were expressed at the same level both in the non-injured and regenerating tissues.

A possible explanation that can be proposed to account for the observed data is that although the analyzed pluripotency factors do not show any large scale over-expression in regeneration, most of them are still expressed at a certain level both in the normal and regenerating tissues. Therefore, it may be hypothesized that this basal level constitutes sufficient environment for dedifferentiation to be triggered by *Myc* upregulation alone. Interestingly, a similar situation has been reported in non-injured and regenerating tissues of lower vertebrates, such as *Danio rerio* and *Xenopus*, where pluripotency markers are never shut off completely under normal conditions. Even though the expression level of these genes remained largely at the same level after the injury, it was hypothesized that this basal expression is neverhteless sufficient to facilitate regeneration upon damage [46].

#### Genes associated with neurogenesis

**Figure 10:**
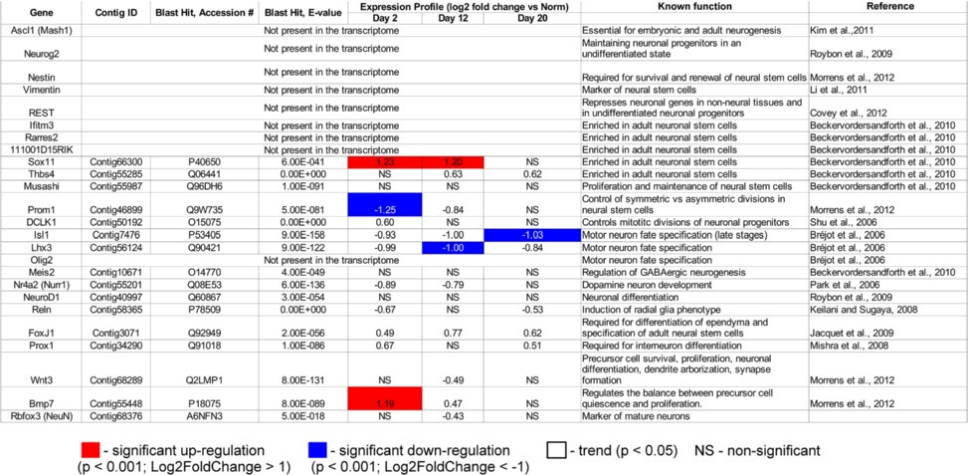
**Expression of neurogenesis-associated genes in regeneration of the radial organ complex in the sea cucumber *H. glaberrima*.** Summary of the digital expression assay and homology search against the UniProt database. Red and blue colors indicate significant (adjusted p < 0.001, more than two fold change in expression level) up-regulation or down-regulation, respectively, in the regenerating tissues as opposed to uninjured organs. NS, no significant changes in expression were observed.

Post-traumatic neurogenesis in the lesioned radial nerve cord involves extensive proliferation of radial glial cells, which then act as progenitors generating both new glial cells and neurons [1,7]. Surprisingly, our functional analysis of differentially expressed genes did not yield statistically significant enrichment of neurogenesis-associated functional categories. The only exception was the enrichment of the category “neuron development” in the set of down-regulated genes during the growth phase on day 12 post-injury (Additional file 6). We then manually searched our databases for homologs of genes known to be implicated in maintenance of neural stem cells and in specification of the neuronal and glial lineages in other animal models (25 genes in total) (see e.g., [47-56]). The results of this analysis are summarized in Figure 10. The most probable explanation why neurogenesis was not a dominant theme in the functional analysis of differentially expressed genes is that most of the neurogenesis-related genes, whose homologs were identified in the transcriptome of *H. glaberrima*, were already expressed in the non-injured radial nerve cord. Their expression level showed some fluctuations in the regenerating tissues, but not above the cut-off threshold (twofold change in expression, adjusted p-value < 0.001), with the exception of up-regulated *Sox11, Bmp7* and down-regulated *Prom1, Isl1,* and *Lhx3*.

To our surprise, homologs of some of the key markers of vertebrate neural stem cells, including nestin and vimentin, were absent from the sea cucumber transcriptome. Likewise, transcripts of some of the important pro-neural factors [55], such as *Mash1* and *Neurog2* were also not detected in either the normal or regenerating animals. This may be due to the fact that the program of post-traumatic neurogenesis in sea cucumbers is not known and, obviously, may not entirely consist of the mechanisms that regulate neurogenesis in vertebrates, whose genes were used as the reference.

#### Prevention of excitotoxic neuronal cell death

**Figure 11:**
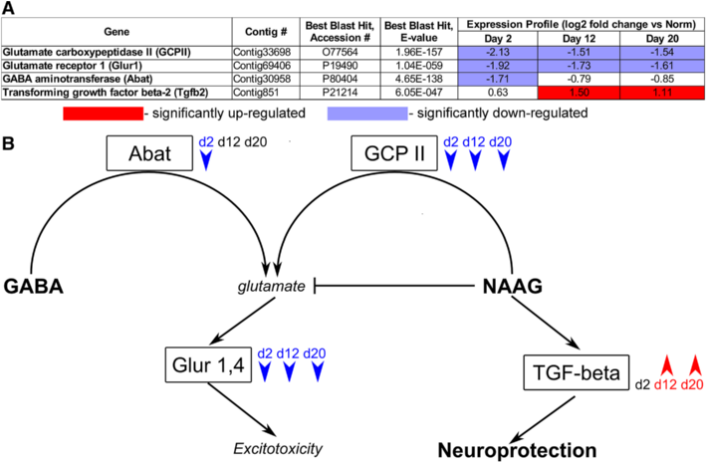
**Suppression of excitotoxicity as a possible adaptation contributing to efficient neural regeneration in echinoderms. (A)** The relevant genes in the transcriptome of the sea cucumber *H. glaberrima*. Their expression levels (relative to the uninjured tissues) and results of homology search against the UniProt database. Red and blue colors indicate significant (adjusted p < 0.001, more than two fold change in expression level) up-regulation or down-regulation, respectively. **(B)** A diagram illustrating a hypothetical mechanism of excitotoxicity suppression in the injured radial nerve cord of *H. glaberrima*. Down-regulation of genes coding for two enzymes, *GCPII* and *Abat*, results in decreased glutamate production. Decreased activity of *GCPII* also leads to accumulation of its substrate, NAAG, which further inhibits release of glutamate from presynaptic terminals, but stimulates production of *TGF-beta*, which promotes neuronal survival. Down-regulation of ionotropic glutamate receptors (*Glur1, Glur4*) reduces the overstimulation of the post-synaptic membrane by glutamate and thus prevents downstream neurotoxic cascades from being triggered.

One of the adaptations that may facilitate neural regeneration in the sea cucumber *H. glaberrima* is inhibition of excitotoxicity (Figure 11). In vertebrates, nervous system trauma results in two types of degenerative processes: the primary damage as the direct result of the injury, which affects the cells in the immediate vicinity of the trauma, and the secondary degeneration, which significantly increases the amount of the affected tissue and is mediated by endogenous mechanisms triggered by the initial injury [57,58]. Excitotoxicity is one of these secondary pathological processes. It involves neuronal death via excessive stimulation by glutamate, whose extracellular concentration increases after CNS injury. Our results suggest that transection of the radial organ complex in the sea cucumber *H. glaberrima* results in significant down-regulation of *glutamate carboxypeptidase II (GCPII)* in the vicinity of the injury (Figure 11). This enzyme hydrolyzes NAAG (N-acetyl-aspartyl-glutamate) to glutamate and N-acetylaspartate. It can be hypothesized that down-regulation of *GCPII* will eventually result in inhibition of NAAG hydrolysis and accumulation of intact NAAG, which is known to further decrease the amount of glutamate released from presynaptic terminals and therefore prevent excitotoxic neuronal cell death [59]. Moreover, NAAG has been reported to stimulate production of important regulators of neuron survival, such as TGF- *β*[59,60]. A sequence strongly related to *TGF- β* is present in the sea cucumber transcriptome. It shows a trend toward up-regulation on day 2 and is significantly up-regulated above the cut-off threshold on days 12 and 20 (Figure 11). On the other hand, the transcripts coding for another enzyme involved in glutamate production (*Abat*) and for the glutamate receptors (*Glur1, Glur4*) are also significantly down-regulated. Suppression of pathways leading to induction of excitotoxic neuronal death and concomitant induction of factors involved in neuronal survival can represent one of the adaptations aimed at minimizing the traumatic impact of the neuronal injury and sparing the maximum possible number of neurons, so that less of them will need to be replenished in the course of subsequent regeneration. A promising further line of research would be to experimentally test if changes in the severity of excitotoxicity would actually affect the extent of tissue damage and the rate of re-connection in the injured radial nerve cord.

## Conclusions

This study was conceived as a first stage in exploring molecular mechanisms behind the observed cellular processes in echinoderm CNS regeneration. In general, functional annotations of the differentially expressed genes corroborate well our previous morphological data, but also open up potential new avenues for future research. As mentioned above, our results point out to the important role of the ECM remodeling in regeneration of the radial complex in the sea cucumber. So far, reorganization of the connective tissue in regenerating echinoderms has received little attention. There have been just two experimental studies addressing this issue directly and in both cases they were focused on regeneration of the digestive tube only [33,34].

Another promising line of future research would be to experimentally test the predictions of this study suggesting the existence of mechanisms suppressing excitotoxicity in the injured CNS. If these mechanisms actually exist, their understanding will be valuable for devising new therapies to prevent excitocytotic neuronal death following, for example, brain and spinal cord injury.

One of the most important outcomes from this study is a predicted list of putative transcription factors, which presumably control differential expression of large groups of downstream genes and thus occupy key positions in regulatory networks controlling regeneration. These genes represent promising candidates for future functional analysis.

Among other interesting findings is that post-traumatic regeneration of the radial nerve cord did not involve large-scale over-expression of pluripotency factors. Many of these genes were already expressed in intact tissues, and only *Myc* showed up-regulation after injury.

We are well aware of the limitations in our study. For example, by design, our functional annotation was dependent on matching the sea cucumber contigs against a well studied proteome from another organism (in this case, mouse). This approach could have led to many potentially relevant sea cucumber-specific sequences being excluded from the analysis. This issue cannot be resolved without carrying out a separate study aimed at characterization of the ‘new’ or ‘unknown’ sequences, which do not have significant homologs in current databases.

The present paper, like most of other high-throughput studies of gene expression, uses mRNA abundance levels to get insight into how changes in gene expression might affect the phenotype of tissues and cells, although it is largely the quantity of the protein that directly determines the phenotype. However, for the time being, transcriptomic approaches are justified by the fact that the current methodologies for direct quantification of protein expression are either less reliable or more laborious and expensive than mRNA-based studies.

Notwithstanding the limitations, the results and predictions reported above are valuable, because they provide a number of clearly defined testable hypotheses, whereas the associated pitfalls and limitations are well known to many researchers working with ‘non-model’ organisms and are not unresolvable in the future.

## Methods

### Sea cucumber collection, maintenance, and radial nerve cord injury

Adult individuals of the brown rock sea cucumber *Holothuria glaberrima* Selenka, 1867 were collected from the intertidal zone of the Atlantic coast of Puerto Rico. The reader is referred to our previous publications for detailed description of the injury paradigm and surgical procedures [1,8,12]. Briefly, the animals were brought to the laboratory and induced to eviscerate (autotomize their viscera) by injecting 0.35 M KCl into the coelomic cavity. The sea cucumbers needed to be eviscerated, because our surgery involved cutting the radial nerve cord from the inside of the body. In order to perform the transection we needed to get access to the inner side of the body wall. We did so by anesthetizing the animals in 0.2% chlorobutanol (Sigma) for 10–30 min and then exposing the coelomic surface of the body wall through the anus by pushing a glass rod against a radial region of the epidermis at the mid-body level. It was only possible after the animals have been induced to autotomize the viscera. *H. glaberrima* does not survive penetrating injuries to the body wall (when there is a direct communication between the coelom and the environment). Nevertheless, it readily regenerates if the injury is made from the coelomic side of the body wall without disrupting the epidermis. We thus cut the radial organs of the mid-ventral radius (including the longitudinal muscle band, water-vascular canal, the radial nerve cord, and the underlying connective tissue), but not the epidermis, with a sharp razor blade. The operated animals were returned to the aquaria and kept at room temperature in well-aerated seawater, which was changed regularly. All experiments were conducted in accordance with the NIH and University of Puerto Rico guidelines for the care and use of laboratory animals.

### RNA extraction and library preparation

Samples for high-throughput sequencing were prepared as previously described [12]. From each regenerating animal, we excised the region of the injury gap (∼3–4 mm wide) plus ∼3 mm of stump (‘old’) tissue on either side of the injury plane. The wet weight of an individual tissue sample was around 10–15 mg. Tissue samples of comparable size and weight were also excised from uninjured animals. During tissue sampling, every effort was made to separate the radial nerve cord from surrounding tissues. However, isolation of the pure nerve cord by surgical means turned out to be practically impossible. Therefore, our tissue samples also consistently contained small amounts of the surrounding connective tissue, an accompanying segment of the water-vascular canal and a stretch of the contractile coelomic epithelium of the body wall because of close anatomical proximity of these structures to the radial nerve cord (Figure 2). For the 454 platform, we generated three non-normalized libraries representing uninjured animals (38 individuals), days 2 and 6 post-injury (63 and 71 animals, respectively), and days 12 and 20 post-injury (62 and 66 animals, respectively). In addition, equal quantities of the above samples were combined to prepare a normalized library. The samples extracted from the regenerating animals on day 6 post-injury were only used for 454 sequencing to increase transcript diversity in the final assembled transcriptome, and were not subjected to sequencing on the Illumina platform (see below). Total RNA was extracted using TRI reagent (Sigma), assessed for quality on an Agilent 2100 Bioanalyzer with the RNA 6000 Nano chips, and subjected to two rounds of poly(A) selection using Poly(A)Purist technology (Ambion). Normalization procedure was performed with a TRIMMER kit (Evrogen) following the manufacturer’s protocol. The normalized cDNA was amplified using Advantage 2 Polymerase Mix (Clontech).

For Illumina sequencing two non-normalized libraries were prepared for each of the four conditions: (i) uninjured radial organ complex (total RNA samples were pooled from 4 and 3 animals for the first and second libraries, respectively); (ii) day 2 post-injury (20 and 19 animals were used); (iii) day 12 post-injury (20 animals were used for each of the libraries); and (iv) day 20 post-injury (15 animals were used for each of the libraries). The final stages in library preparation and sequencing were performed by sequencing service providers at the DNA Facility of the University of Iowa (Genome Sequencer FLX System, Roche) and the Genome Sequencing and Analysis Core Facility of the Duke Institute for Genome Sciences and Policy (Illumina Genome Analyzer IIx, Illumina). Raw sequencing reads from both the 454 and Illumina platforms were deposited at NCBI Sequence Read Archive (SRA) under accession number NCBI:SRA051990 [61].

### De novo assembly pipeline

The first round of filtering/cleaning of 454 reads was performed with SeqClean [62] and included removal of synthetic adaptor/primer sequences used in library preparation and screening for *E. coli* contamination (GenBank:U00096). The reads were then processed with the standalone version of PRINSEQ tool (v 0.14.2) [63] with the following filtering parameters: minimum length — 60 bases, maximum length — 1100 bases (twice the mean length), maximum allowed percentage of N’s – 1%, minimum mean quality — 17. Also, exact duplicates and reverse complement exact duplicates were removed.

Raw Illumina sequencing reads were quality checked using the FastQC tool (v 0.9.1) [64] and then trimmed using FASTX-Toolkit (v 0.0.13) [65] to remove Illumina adapters, discard bases with quality < 20, and reads shorter than 32 bases.

We applied a complex pipeline to assemble the pooled reads from all libraries into a single reference contig set (Figure 3). First, we used MIRA 3.2.1 [66] to assemble all normalized and non-normalized 454 reads. The Illumina reads were assembled with Velvet (v.1.1.03) [10] and Oases (v.0.1.21). Separate assembly runs were performed at different kmer lengths (31 to 55). Cleaned 454 reads were introduced into each of these runs to improve the assembly. The contigs longer than 100 bp resulting from the Velvet/Oases assemblies were combined together with 454 contigs for the final assembly with CAP3, to produce what we refer to as the reference library. The files containing the contigs, their annotations, and expression values were parsed and analyzed using custom written scripts, which are available from the authors by request.

### Differential gene expression in regeneration

For digital expression assay based on RNA-seq data, we used Bowtie 2 [67] to map Illumina reads to the contigs of the reference library. The raw read counts for each of the eight Illumina libraries (four conditions, two biological replicates per condition, see above) were used as input to the DESeq R package [16] to perform pairwise differential expression analysis between the intact and regenerating animals. The estimateSizeFactors function of the DESeq package was used to normalize gene counts. The resulting *P* values were adjusted for multiple testing with Benjamini-Hochberg procedure. Genes with an adjusted *P* value < 0.001 and a fold change greater than 2 were considered differentially expressed.

In order to validate the changes in gene expression determined by RNA-seq, we selected 21 genes with different levels of transcript abundances for real-time PCR analysis. Poly(A) RNA was extracted as described above. PCR primers were designed using Primer Premier 5.0 software (PREMIER Biosoft International). Their sequences are shown in Additional file 10. RNA was reverse transcribed with random hexamer primers and SuperScript II reverse transcriptase (Invitrogen). Template cDNA was diluted 10-fold or 100-fold and used at 2 *μ*l per 25 *μ*l of PCR reaction with Brilliant SYBR Green Master Mix or Brilliant II SYBR Green Master Mix (Agilent) following the manufacturer’s protocol. Real-time PCR reactions were run on Mx3005P qPCR System (Stratagene). The reactions were performed on three independent samples per condition (biological replicates). Each sample was analyzed at least twice, making sure that the difference between technical replicates was less than 0.5 Ct [68]. PCR efficiencies were evaluated by running five 10-fold dilutions of the cDNA template in a PCR reaction and were considered acceptable if the corresponding slope values determined by the MxPro QPCR software (Strategene) lay between -3.2 and -3.5 and the R ^2^ was above 0.98. All expression values were normalized relative to the ’normalization factor’ calculated with the geNorm Visual Basic Application for Microsoft Excel [69] from the expression of values of four genes (*Rpl18a, Atp6l, Eef2,* and *Sod*), which were identified among the least changing transcripts across the experimental conditions by RNA-seq.

### Annotation

The assembled contigs of the reference library were used as input for the BLASTX homology search. Initially, the sea cucumber sequences were compared with the Swiss-Prot database with a cut-off significance threshold set at 1e-6. Those contigs that lacked matches were then subjected to a second round of BLASTX search against the larger NCBI non-redundant database. In addition, the sea cucumber transcriptome was also annotated versus the NCBI’s collection of the sea urchin predicted protein sequences [14] by performing reciprocal best BLAST hit analysis (with a threshold e-value < 1e-6).

Functional annotation of differentially expressed genes was performed with DAVID Gene Ontology web server [18]. In order to be able to use this tool, we matched all 70,173 contigs of our assembled transcriptome to the non-redundant reference proteome of the mouse [70], release 2012_05 using BLASTX with the cut-off e-value of 1e-6. Overall, our assembled contigs showed significant homology to 8,522 mouse genes. We then submitted the annotated lists of differentially expressed genes as an input to DAVID and analyzed them against the background of all annotated genes of our reference library. For pathways of interest, KEGGanim [71] was used to generate diagrams showing changes in expression level of individual genes. This approach allows to observe expression dynamics in the context of specific pathway interactions.

### Expression profile clustering

Unsupervised gene expression profile clustering of differentially expressed genes (i.e., the genes showing more than two-fold change in expression with adjusted *P* < 0.01) was performed using AutoSOME 2.0 software [20]. Prior to clustering, the original count data were subjected to variance stabilization transformation in the DESeq package. The parameters were set as suggested by the authors of the program (running mode: precision, number of ensemble runs: 500, *P*-value threshold: 0.05). Unit variance, median centering (rows), and sum squares (both rows and columns) normalization procedures were applied. Eight clusters containing more than 100 contigs were visualized on a heatmap (Figure 7) and considered for further analysis.

### Identification of putative regeneration-associated transcription factors

Over-represented transcription factors associated with co-expressed genes were predicted with oPOSSUM v3.0 software [21]. Lists of gene identifiers corresponding to each of the cluster identified by AutoSOME were used as input. The list of gene identifiers corresponding to the entire reference library was used as the background set. The JASPAR CORE collection was used as a set of transcription binding site matrices. The conservation cutoff and the matrix score threshold were left at their default values of 0.4 and 85%, respectively.

In order to select potentially relevant transcription factors, we followed the suggestions of the software’s authors and plotted the Z-score against Fisher score for the transcription factors associated with the genes in each of the cluster. The genes, which showed clear segregation of the scores, were considered for further analysis, if changes in their expression were significant (adjusted *P* < 0.05) at least at one of the three analyzed time points. If the expression level of the putative transcription factor changed in the same direction as the median gene expression in the gene cluster, this transcription factor was considered a transcriptional activator. If the change in expression was in the reverse direction, the transcription factor was considered a transcriptional repressor.

## Availability of supporting data

Raw sequencing reads supporting the results of this article are available in the NCBI SRA repository [61]. The contigs of the reference library, representing the transcriptomic diversity in the normal and regenerating radial organ complex of *H. glaberrima* are available in the LabArchives notebook [11]. Other data sets are included within the article and its additional files.

## Abbreviations

ECM: Extracellular matrix; LTR: Long terminal repeat; ORF: Open reading frame; *P*_*adj*_: Adjusted *P*-value; RNA-seq: Next-generation sequencing of expressed mRNA; TF: Transcription factor.

## Competing interests

The authors declare that they have no competing interests.

## Authors’ contributions

VSM, ORZ, and JEGA conceived the study, analyzed the data, and interpreted the results. VSM and ORZ carried out experimental procedures. VSM drafted the manuscript. VSM, ORZ, and JEGA finalized the manuscript. All authors read and approved the final version of the manuscript.

## Supplementary Material

Additional file 1 Best BLASTX hits obtained in similarity searches versus the Swissprot and NCBI non-redundant protein databases. Click here for file

Additional file 2 **Reciprocal best blast hits between the *H. glaberrima *contigs and NCBI’s collection of predicted proteins of the sea urchin *S. purpuratus*.** The data are represented in a tabular file with the following columns: *H. glaberrima* contig ID; *S. purpuratus* protien ID; E-value (BLASTX search: *H. glaberrima* contigs vs sea urchin proteins); bit score. Click here for file

Additional file 3 **Correlation of gene expression values between biological replicates for each of the four conditions (normal animals, day 2 post-injury, day 12 post-injury, and day 20 post-injury).** Each dot indicates normalized read count returned by the DESeq package. The straight red line is a linear regression fit of data points. Pearson’s product-moment correlation coefficient is indicated for each comparison. Click here for file

Additional file 4 **Frequency histogram of fold change in expression level of differentially up-regulated (left column) and down-regulated (right column) contigs at three stages of the radial organ complex regeneration.** The total number and percentage of contigs showing changes in expression between 2- and 4-fold relative to the normal animals are indicated next to each plot. Click here for file

Additional file 5 Fold change ratios (relative to uninjured animals) for 21 genes as determined by RNA-seq and real-time qRT-PCR. Click here for file

Additional file 6 **Functional annotations of differentially expressed genes at three time points of radial organ complex regeneration.** Differentially expressed contigs were analyzed for significantly enriched annotation terms, which were clustered using DAVID. Click here for file

Additional file 7 **AutoSOME output.** Tabular file containing cluster label, cluster confidence and expression level for each data point. Click here for file

Additional file 8 **Differentially expressed cancer-related genes (Pathways in cancer, KEGG) at three time points (days 2, 12, and 20 post-injury) of radial complex regeneration in the sea cucumber.** The gene expression data were mapped to KEGG pathways using the KEGGanim web tool. Click here for file

Additional file 9 **Differentially expressed genes of the Focal Adhesion KEGG pathway at three time points (days 2, 12, and 20 post-injury) of radial complex regeneration in the sea cucumber.** The gene expression data were mapped to KEGG pathways using the KEGGanim web tool. Each rectangle represents either a single or multiple genes. When a rectangular node on the graph represents several genes with significant changes in their expression values, a list of genes is provided with color coding for up-regulation (red) or down-regulation (blue) relative to the expression levels in intact animals. Click here for file

Additional file 10 Primers used for quantitative real-time PCR. Click here for file
