# Supplementary figures and images for: Transcriptomic changes during regeneration of the central nervous system in an echinoderm
Source: BMC Genomics. 2014 May 12;15:357. doi: 10.1186/1471-2164-15-357 (PMC4229883; doi:10.1186/1471-2164-15-357)

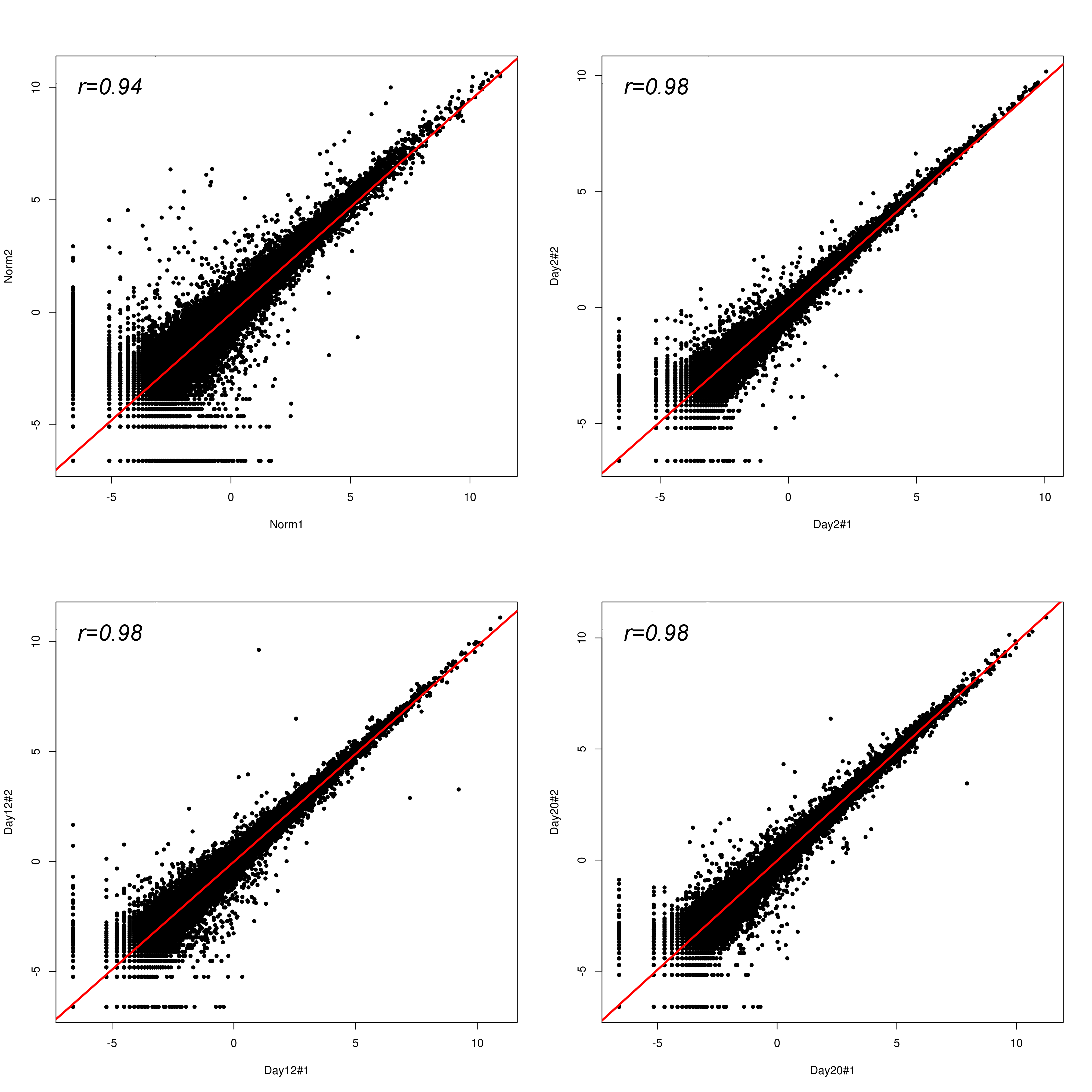

Supplement: Additional file 3 — Correlation of gene expression values between biological replicates for each of the four conditions (normal animals, day 2 post-injury, day 12 post-injury, and day 20 post-injury). Each dot indicates normalized read count returned by the DESeq package. The straight red line is a linear regression fit of data points. Pearson’s product-moment correlation coefficient is indicated for each comparison. [file 1471-2164-15-357-S3.tiff]

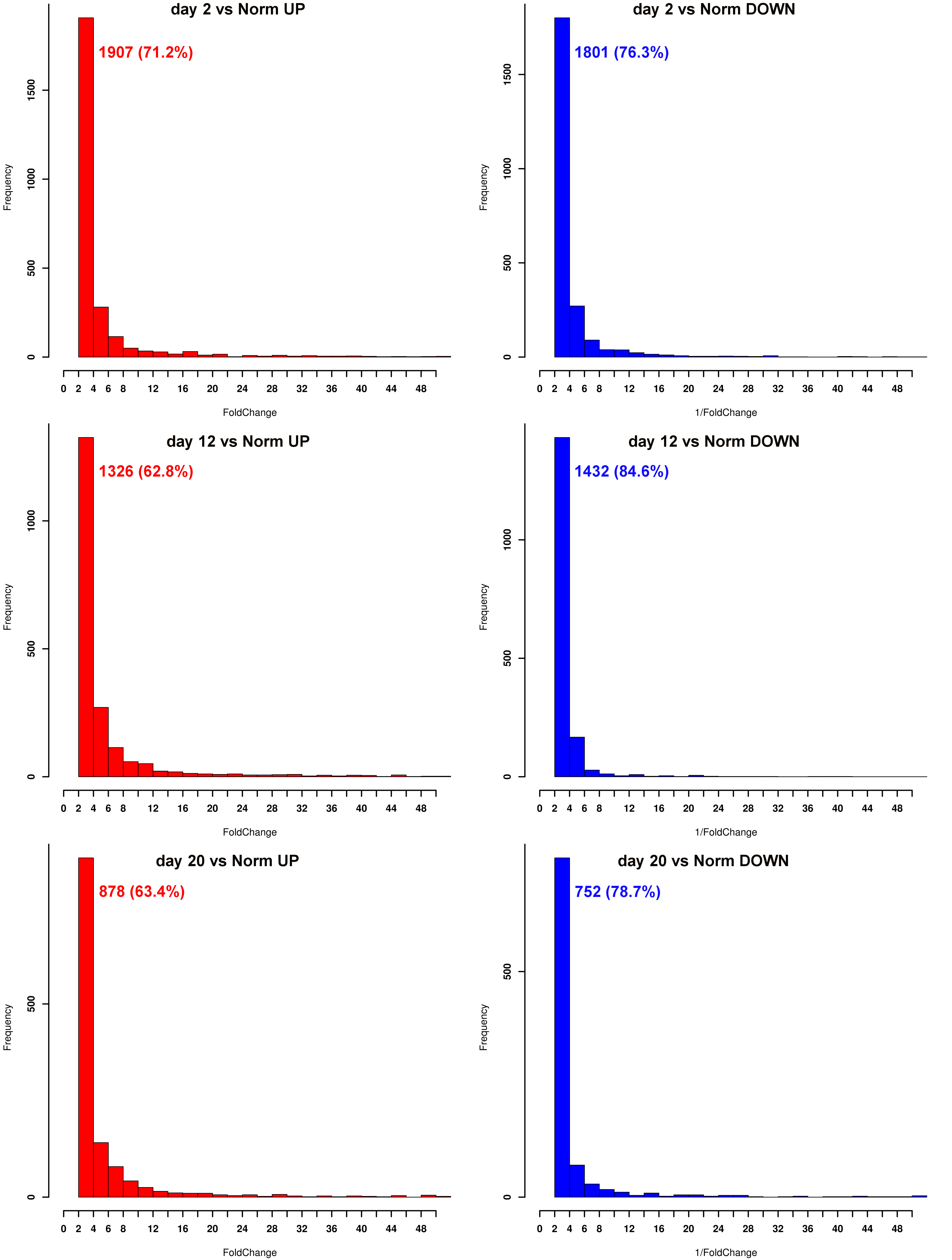

Supplement: Additional file 4 — Frequency histogram of fold change in expression level of differentially up-regulated (left column) and down-regulated (right column) contigs at three stages of the radial organ complex regeneration. The total number and percentage of contigs showing changes in expression between 2- and 4-fold relative to the normal animals are indicated next to each plot. [file 1471-2164-15-357-S4.tiff]

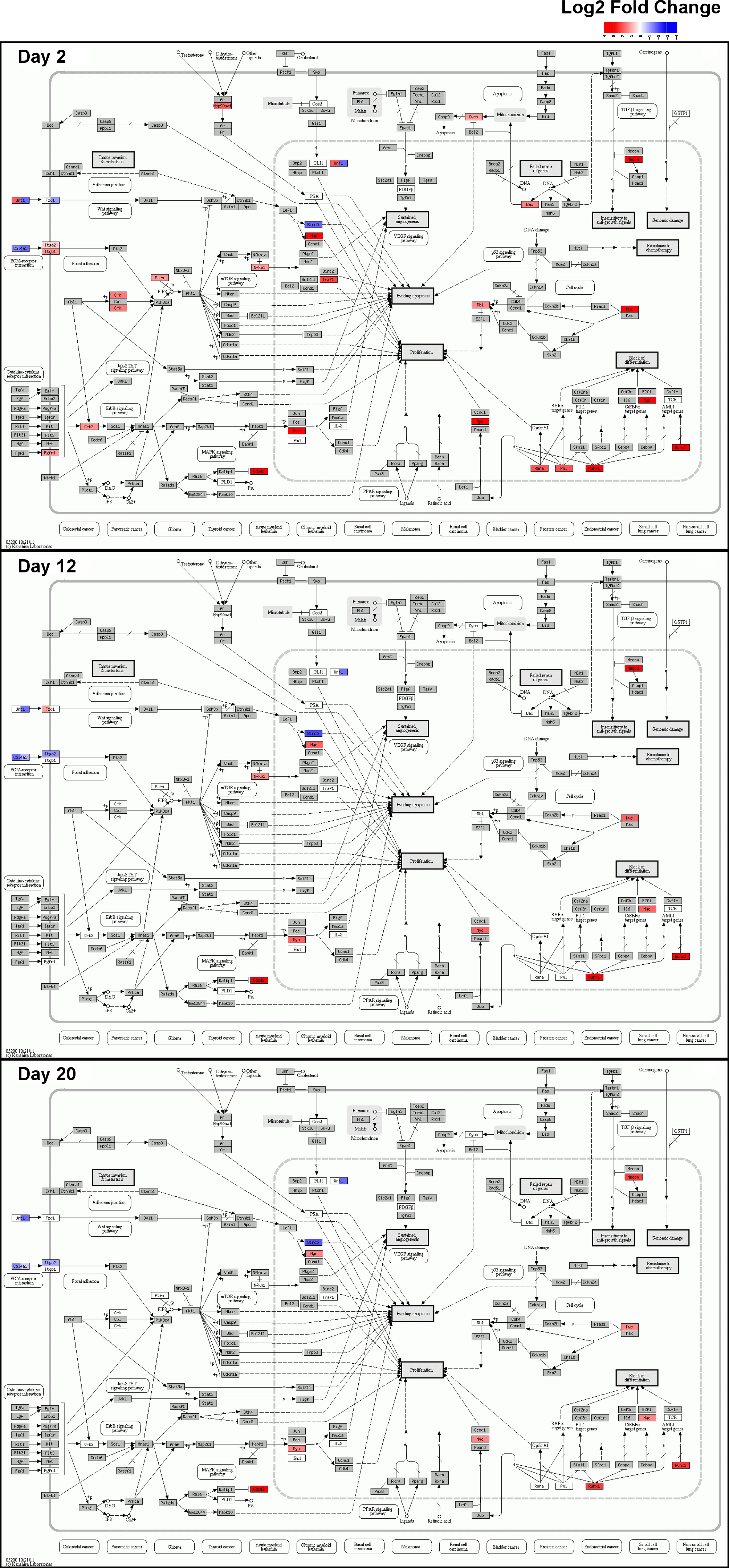

Supplement: Additional file 8 — Differentially expressed cancer-related genes (Pathways in cancer, KEGG) at three time points (days 2, 12, and 20 post-injury) of radial complex regeneration in the sea cucumber. The gene expression data were mapped to KEGG pathways using the KEGGanim web tool. [file 1471-2164-15-357-S8.tiff]

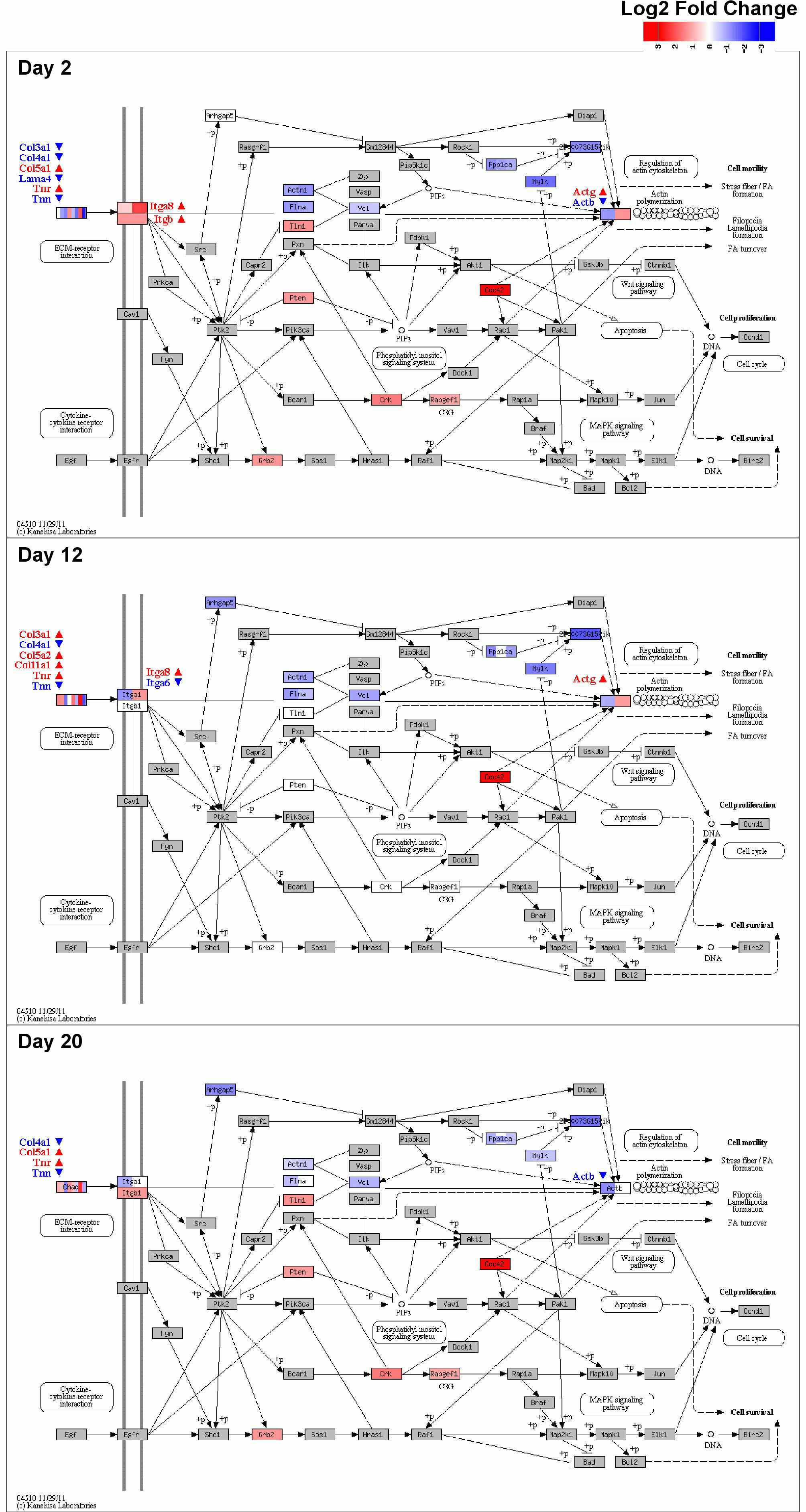

Supplement: Additional file 9 — Differentially expressed genes of the Focal Adhesion KEGG pathway at three time points (days 2, 12, and 20 post-injury) of radial complex regeneration in the sea cucumber. The gene expression data were mapped to KEGG pathways using the KEGGanim web tool. Each rectangle represents either a single or multiple genes. When a rectangular node on the graph represents several genes with significant changes in their expression values, a list of genes is provided with color coding for up-regulation (red) or down-regulation (blue) relative to the expression levels in intact animals. [file 1471-2164-15-357-S9.tiff]
