# Supplementary material for: Transcriptomic changes during regeneration of the central nervous system in an echinoderm
Source: BMC Genomics. 2014 May 12;15:357. doi: 10.1186/1471-2164-15-357 (PMC4229883; doi:10.1186/1471-2164-15-357)
Supplement: Additional file 10 — Primers used for quantitative real-time PCR. [file 1471-2164-15-357-S10.pdf]

Primers used for real-time qPCR

Transcript	Forward primer (5' → 3')	Reverse Primer (5' → 3')
Myc	CCTACAGCCGTCTTTCCATACC	TCCCTGCCATACTGAGATCCA
Nfix	TTGAGACAGGCAGATAAAGTATGGA	CACAATGATGGGTTCGCACA
Timp1	GCTGTATTCATTAGTCCACTTCCA	GGGATTGCTACTACAACCCATT
Gypsy-1_Hg	GCTGTTCCGCACTATCATTG	GCTGTTCCGCACTATCATTG
Gypsy-2_Hg	ATGGAAACGCAGATGCTCTA	CGGAAGTGGTTCTTGTGGTAA
LINE1	AAACCAGACGGGCTTCCTTC	GAATGAAATTCCAGTCTATGGTATCG
Runx1	TCTTGCCAGGGTCTCCATCA	CGGTCTTCCAAATCCAACTCAC
Echinoidin	AAAGTGTGTGTCCTTGTCATACAGC	TGTAGGTGGAACTAGATCCGTCAC
Notch 1	GCCAACAATGAGCGAGGATAG	AGGGATACCTCCAAGTGACATATTC
Stxbp 1	GGGTATTCCACTCAGTTACGATTG	GGGACAATGTTTCTCATTTGGTCTT
Klf2	GCCTCACATGAGCCTCCTGAC	GGTGGAATTACTGCGTTGTTCTC
Sox2	GTGCCATTTGCCTTCTCTTACC	GAGTCCACAGCCCATCGTTTC
Oct1	TCAGGGTTGCTTTGGAGAAGAG	GGCAGCATCATAGAGGGAGGAT
Notch 2	TATATGCCGAAACACCGATGA	AACAAGGTTCCGCTTTGACG
Cenps	TATGGAAGAACAAAGCGATGGA	TTGCTTGTATGTAGCCTCAGAAATG
Similar to predicted protein XP_001628344	TCAAGTACACTAACTGTTCCCATCT	AACTGAAATCCCACCACCC
Cntn5	CCTTCTCCAAGCCAAAGTCG	CCATCAGACCCACCGCAA
Srpx2	AAAGGTGCGACCTGTACTTTCAC	GATACAATTCGGGATTTCTGTGTT
Gypsy-20_Hg	GGGGAATACTAGACCTACAGAATGG	GGTGGAATGGTAACAGTTTCAGC
Jockey mobile element	CTGAGGGGTCAAGTAGAAGGGA	CTTAAACCTCCTGAAGACAAACCA
Neurotrypsin	GGATGATAATGATGCCACGGTA	CACCGATACTTGTTTCTGAACCAG

Reference genes

Transcript	Forward primer (5' → 3')	Reverse Primer (5' → 3')
Rpl18a	AACAAGGAGACCAGCTACCAACT	ATGTAATTCCAACCCACTTTCAGG
Atp6l	CGGAGCAGGACTTAGTGTCG	TTCCAACAAACAAACGAGGCT
Eef2	TGAAGGTCCACGAGACGATCC	CAGAGAAGACACGACCAAAGGC
Sod	CTCACTAAGCCCTGAAACGACA	ACGGTTCTTCGCTTTCTACTTCA
